# Where can care workers go to the toilet? The right to working conditions that ‘respect health, safety and dignity’

**DOI:** 10.1177/17579139241270802

**Published:** 2025-09-23

**Authors:** S Rutter, C Needham

**Affiliations:** Information School, University of Sheffield, The Wave, 2 Whitham Road, Sheffield S10 2AH, UK; School of Social Policy, HSMC Park House, University of Birmingham, Edgbaston, Birmingham B15 2RT, UK

## Abstract

This Feature article draws attention to the challenges care workers who work in the community face accessing hygiene facilities such as toilets and the impact this has on their health and working conditions. There is public awareness of the challenges faced by delivery drivers but the conditions faced by care workers are not recognised as frequently.

The challenges delivery drivers and warehouse workers face accessing toilet facilities have been widely reported in the media (e.g. Oldfield^1^ and Pope^2^). Moreover, a report by the royal society for public health (RSPH)^3^ considers the decline of public toilets including the impact on workers on the move such as tourist guides, drivers and postal workers. Less reported are the challenges faced by care workers who work in the community and the impact these have on their health and working conditions.

The importance of providing hygiene facilities is widely recognised in workplace legislation. For example, the UK Health and Safety Executive requires that ‘employers have to provide [toilet and washing] facilities suitable for any worker’.^4^ However, providing facilities when workers are working away from employer-owned facilities can be challenging. For some occupations, such as construction workers and delivery drivers, this can be overcome to some extent by providing portable toilets and requiring that worksites visited provide access. However, for care workers visiting clients’ homes, these are not viable solutions.

Rutter et al.^5^ have found that there is little or no provision for care workers to access hygiene facilities for their personal needs. While they are working in the community, they must seek permission to access hygiene facilities in clients’ homes or travel to hygiene facilities that are in decline and difficult to find. The facilities they are able to access can be low quality (e.g. no soap and dirty towels) and may cost them money to access (e.g. an entrance fee, buying a drink to become a customer), and privacy is a concern particularly if the care worker is taking a client out and cannot leave them unattended. When they are unable to access facilities, care workers restrict what they drink and eat, ignore urges or soil their clothes. This has serious implications for their health and dignity.

**Figure:**
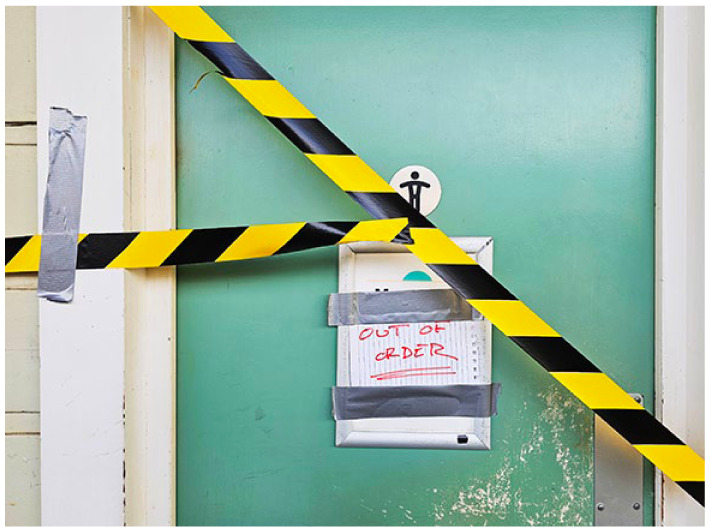


According to Article 31 – Fair and Just Working Conditions of the EU Charter of Fundamental Rights,^6^ ‘every worker has the right to working conditions which respect his or her health, safety and dignity’. While access to toilets is not usually considered a measure of working conditions (see for example working conditions as described by Eurofound and International Labour Organisation),^7^ we argue that access to hygiene facilities is an important indicator. Beyond the importance of good working conditions for the care workers themselves, there are further implications as good working conditions contribute to the success of organisations,^7^ and care workers’ working conditions are widely recognised as determinants of quality care.^8^

Given that people who menstruate and older people^9,10^ may be particularly adversely affected by lack of access to toilets and other hygiene facilities, and there is a high proportion of (often older) women in the care workforce,^11^ it is particularly concerning that care workers struggle to access toilets. Arguably this is an occupational health equality issue,^12^ whereby workers who are more at risk of occupational illness and injury are working in environments that exacerbate this risk.

The social care sector is experiencing a recruitment crisis.^13^ Poor working conditions can lead to difficulties in care worker recruitment,^8^ and it is worth considering whether access to toilets could be a factor here too. The point is commonly made that care workers can earn more working in a supermarket (e.g.).^14^ What is not noted is that in a supermarket, they would also have access to a staff room and toilets on site.

We have focused here on social care workers, who usually do not have an accessible base that they can return to between seeing clients. It is important to note that lines between occupational roles are blurring, especially between health and social care. Social care workers are taking on delegated health roles, and there are increased moves to locate acute care out of hospitals, for example, with hospital at home and virtual wards.^15^ Social care workers are among one of the groups now recognised to be part of the public health workforce in the community.^16^ Needham et al.^17^ have written about the emotional labour of ‘role extending’, as people acquire new roles on top of their core occupations. We might also pay attention to how the blurring of roles is changing the places and conditions in which people work, and their access to essential facilities such as toilets.

When social care services are being commissioned, and health services are being moved into the community, many factors are considered but not the ability of workers to access toilets and other hygiene facilities. Given the importance of this to safety and dignity at work we call for more attention to be paid to this issue. Over 50 local authorities have signed up to Unison’s Ethical Care Charter^18^ which relates to pay and conditions for care staff; these are undeniably important. But let us remember that what makes a good working day for all of us is not just pay, conditions and safety but also confidence that we can access a toilet when we need to.
